# A Spotted Child

**Published:** 1847-05

**Authors:** 


*A Spotted Child.*—A foundling child was brought to the New York Alms House on Saturday last. It was a little girl about sixteen months of age. About one half of its body was of a fair complexion, and the remainder of a rich copper color, and the dark spots were all covered with a long white hair. In the centre of its forehead was a round dark spot, and the same hue covered the breast and legs.—*Ibid.*

